# Registry-recorded stroke and transient ischaemic attack diagnoses in Saudi Arabia, 2016–2025: a national descriptive analysis

**DOI:** 10.3389/fstro.2026.1917036

**Published:** 2026-10-27

**Authors:** Aisha M. Alshehri, Mohammed AlJawadi, Abdulrahman M. Alsheikh, Malak E. Aloufi, Yahya A. AlMazni, Abrar M. Alasmari, Amani O. Al Fadhli, Meral M. Homoud, Wasaal I. Alquaydhib, Omar A. Alsuhaibani, Sarah F. Almuqati, Shatha M. Alshaibi, Abdulaziz M. Eskandarani, Fatima Y. Al Slail, Mubarak Aldawsari, Muaddi F. Alharbi, Mariam M. AlEissa, Shaker A. Alomary, Abdullah Assiri, Mohammed AlAbdulali

**Affiliations:** 1 Population Health Observatory, Ministry of Health, Riyadh, Saudi Arabia; 2 Council of Health Insurance, Riyadh, Saudi Arabia; 3 Lean Business Services, Riyadh, Saudi Arabia; 4 Scientific Research Office, Assistant Ministry of Health for Health Services, Riyadh, Saudi Arabia; 5 College of Medicine, Alfaisal University, Riyadh, Saudi Arabia; 6 Public Health Laboratory, Public Health Authority, Riyadh, Saudi Arabia; 7 Research Centre, King Khaled Eye Specialist Hospital (KKESH) Research Centre, Riyadh, Saudi Arabia; 8 KAPSARC School of Public Policies (KSPP), King Abdullah Petroleum Studies and Research Center (KAPSARC), Riyadh, Saudi Arabia

**Keywords:** comorbidities, epidemiology, healthcare utilization, population health observatory, prevalence, risk factors, stroke

## Abstract

**Background:**

Stroke is a major cause of death and disability globally, and its prevalence is growing as modifiable risk factors become more prevalent and the population ages. In Saudi Arabia, evidence is mainly from hospital or local data, and national descriptions of cerebrovascular diagnoses recorded in routinely collected health data remain limited in Saudi Arabia.

**Objective:**

This study aimed to describe the cumulative registry-recorded burden of adults with a qualifying stroke-related or transient ischemic attack (TIA) diagnosis in the Population Health Observatory (PHO) in Saudi Arabia, using linked national health data from 2016 to 2025. This includes the registry-recorded burden, demographics and characteristics, comorbidities and healthcare utilization.

**Methods:**

We conducted a national retrospective descriptive analysis of de-identified aggregate outputs from linked PHO data for 2016–2025, a descriptive statistical analysis of 68,634 stroke-diagnosed individuals in the Kingdom of Saudi Arabia. The observational study was conducted using PHO data from 2016 to 2025.

**Results:**

The PHO identified 68,634 unique adults with a qualifying cerebrovascular diagnosis. Stroke burden was disproportionately concentrated in males (64.45%) and in both genders aged 60–69 years (23.27%). The accumulated count corresponded to 0.26% of the 2025 adult population; this is a crude descriptive ratio, not prevalence. Najran had the highest crude regional ratio at 0.38%. BMI was missing/invalid for 39,481 (57.52%). Among 29,153 with valid BMI, 10,494 (36.00%) were overweight and 9,930 (34.06%) were obese. The PHO low-multimorbidity category contained 42,882 individuals (62.48%); it is an administrative stratum, not a validated cardiovascular-risk score; most visits were to outpatient departments.

**Conclusion:**

Saudi Arabia's recorded stroke burden is concentrated among older males. The findings describe patterns captured by the PHO and should not be interpreted as prevalence, incidence, or causal associations, which indicate the need for better prevention, management of chronic diseases and a robust national surveillance system to inform policies based on evidence to support Saudi Vision 2030.

## Introduction

Stroke remains a leading cause of death and long-term disability worldwide. Global analyses estimate approximately 11.9 million incident strokes annually, with burden influenced by population growth, aging, and modifiable vascular risk factors. Approximately 65.3% of strokes are ischaemic, typically caused by arterial blockage, while the remainder are haemorrhagic, resulting from arterial rupture (G. B. D. 2019 S. Collaborators, 2021; Feigin et al., 2024; Hung et al., 2023). Over the past 30 years, the global burden has intensified, driven not only by population growth and aging but also by largely preventable risk factors such as poorly controlled hypertension, type 2 diabetes, obesity, dyslipidaemia, and sedentary behavior (Feigin et al., 2022). Despite advances in prevention, acute management, and rehabilitation over the past two decades, stroke remains a major burden on healthcare systems, particularly in low- and middle-income countries, where most strokes now occur (G. B. D. 2019 S. Collaborators, 2021; Feigin et al., 2022). The parallel rise in cardiovascular risk factors worldwide, combined with an aging population, means the absolute burden of stroke will continue to grow even as age-standardized rates may decline slightly (WHO, 2025; Mahfouz et al., 2025).

Saudi Arabia has experienced a major epidemiological transition in which non-communicable diseases account for a growing share of morbidity. Over the last 30 years, non-communicable diseases have become the Kingdom's leading cause of illness, including stroke. Increasing urbanization, changing dietary habits and increased obesity, diabetes and hypertension have all contributed to the population's risk of stroke (Asirvatham and Marwan, 2014). To address this challenge, Saudi Arabia's Vision 2030 has initiated a comprehensive health sector transformation specifically intended to emphasize the importance of prevention, early warning and population health management rather than the more traditional, hospital-based approach to healthcare (Aleissa et al., 2026; MOH, n.d.).

Published Saudi stroke data have largely originated from individual hospitals, regions, or selected populations, limiting national generalisability (Asirvatham and Marwan, 2014; El-Hajj et al., 2016; Alqahtani et al., 2020). None of the published studies provides a complete picture of the distribution of stroke by age, sex and geographic location in the Kingdom. Regional studies conducted in the Middle East and North Africa (MENA) have shown significant variations in the burden of stroke and risk factors across the region. However, there is still a need for a comprehensive, representative dataset in Saudi Arabia to guide its response (Jaberinezhad et al., 2022; Alenzy et al., 2026).

The Population Health Observatory (PHO) integrates national health-system data to support population surveillance, service planning, and policy development and was established to fill this gap (Barker-Collo et al., 2015). Importantly, the PHO also considers social determinants of health (SDoH), allowing for analyses that look beyond individual clinical data to encompass the wider determinants of health in Saudi Arabia (Alosaimi et al., 2025; Aleissa et al., 2025). It gathers national health system data to allow monitoring of diseases at a scale and level of detail that is impossible with any single study, monitoring trends, estimating the burden of disease and providing the sort of population-level data that can inform healthcare policy. To achieve the goals of Vision 2030, customized innovation is needed to improve life expectancy in Saudi Arabia (Alosaimi et al., 2025; Aleissa et al., 2025).

In this context, the current study draws on PHO data to provide the first national snapshot of stroke in Saudi Arabia. In particular, we sought to accomplish the following: (i) estimate national registry-recorded stroke burden, (ii) quantify adults with qualifying stroke-related or TIA diagnoses; describe demographic distribution, BMI, PHO multimorbidity strata, and encounters. The study reports registry capture, not prevalence or incidence. (iii) describe the metabolic and other clinical risks of those affected and (iv) describe the utilization of health services among this population. Our findings can help guide public health policy, support health service planning and provide actionable information to inform Saudi Arabia's response to the country's growing burden of non-communicable disease.

## Methodology

### Ethics

Ethical approval 25-108 M, registration H-01-R-009, with the use of anonymised secondary PHO data, and a waiver of individual informed consent. The data used in this study were anonymised to ensure the absence of identifiable patient information. Secondary data were obtained through the PHO portal, consistent with the data center at the Ministry of Health (MOH), in compliance with national data governance standards.

### Study design

This study analyzed individuals diagnosed with stroke. This national retrospective descriptive study used de-identified aggregate PHO outputs for 2016–2025 from Raqeem, NPHIES, Wasfaty, and the National Data Bank. Raqeem represents a unified electronic health record (EHR) of MOH primary healthcare centers (PHCCs). Nphies covers the clinical data of private and public hospitals, while Wasfaty provides electronic prescriptions and diagnostic records across the entire Kingdom.

The National Data Bank integrates all previous registries and more to create a national-level population registry, enabling in-depth analysis of the entire adult population of Saudi Arabia at the clinical, social, service utilization, and demographic levels. Independent and dependent variables were used in the study. The variables were split into three groups. A. Demographic characteristics: age (categorical groups), gender (male/female), nationality (Saudi/non-Saudi), residence (urban/rural), vital status (alive/dead), insurance status (insured/uninsured) and region (Riyadh, Makkah, etc.). B. Clinical characteristics such as body mass index (BMI), multimorbidity (obese, overweight, healthy weight and underweight), PHO multimorbidity strata (low, rising, high, and critical), defined according to the number of additional documented comorbid conditions, and survivability windows (5 years, 1 year, 5 months and 1 month). C. Health-care utilization: visits made (1–2, 3–4, 5–6, 7 or more), outpatient visits, emergency room visits and hospital admissions. The PHO multimorbidity strata were defined according to the number of documented comorbid conditions in addition to the qualifying stroke/TIA diagnosis: low, no additional condition; rising, 1–2 additional conditions; high, exactly 3 additional conditions; and critical, 4 or more additional conditions. These categories were mutually exclusive and represent administrative multimorbidity strata rather than a validated cardiovascular-risk prediction score.

One of the significant national platforms that can be used to enhance the healthcare infrastructure is the PHO (Al Eissa et al., 2026), which was created within the framework of the Population Health Department of the Saudi Ministry of Health. It enables the integration of diverse health data to support the overall tracking of disease dynamics, the assessment of health burdens, and the development of evidence-based policies. The PHO enables effective national-level surveillance of population health patterns and is used to inform health planning strategies through improved data integration and analytical techniques. Moreover, it can be used to add information about healthcare systems and SDoH, which can result in in-depth population-level analyses that cannot be achieved with conventional surveillance systems.

This study is reported in accordance with the REporting of studies Conducted using Observational Routinely-collected health Data (RECORD) statement (Benchimol et al., 2015), an extension of STROBE (von Elm et al., 2007) for routinely collected health data. An accurate reporting summary is provided in Supplementary Table S1.

### Cohort definition and data sources

National unified health registries were utilized in this study using the PHO to extract and analyse the individual health records of over 68,000 individuals spanning the period of 2016 to 2025. The diagnosis of stroke was identified using stroke-related International Classification of Diseases (ICD10) codes: I61 intracerebral hemorrhage; I62 other non-traumatic intracranial hemorrhage; I63 cerebral infarction; I64 unspecified stroke; and G45.8 transient ischaemic attack. The study participants included adults aged >=18 years with a qualifying registry-recorded diagnosis between 1 January 2016 and 31 December 2025 with a stroke-related International Classification of Diseases, Tenth Revision (ICD-10) code. The study cohort included ischemic stroke, intracerebral hemorrhage, other non-traumatic intracranial hemorrhage, unspecified stroke, and transient ischemic attack (TIA) to provide a comprehensive assessment of the national burden of cerebrovascular disease recorded within the Population Health Observatory. Although TIA is clinically distinct from stroke, it shares common vascular risk factors, prevention strategies, and referral pathways and is widely recognized as an important predictor of subsequent stroke. Therefore, it was included in the overall descriptive analysis. Future studies will perform subtype-specific analyses to investigate differences in epidemiology, healthcare utilization, and outcomes across cerebrovascular disease subtypes (Easton et al., 2009; Kleindorfer et al., 2021). The descriptive analysis of annual counts of newly registry-recorded stroke/TIA diagnoses was restricted to 2020–2025 because registry completeness, electronic health record implementation, and national data linkage were considered more mature during this period. Because registry completeness, electronic health record implementation, and national data linkage reached sufficient maturity during this period to permit more reliable identification of newly registry-recorded stroke/TIA diagnoses. Earlier years (2016–2019) were not included in the incidence analysis because progressive expansion of the PHO database during the initial implementation phase may have resulted in incomplete ascertainment of incident cases (Al Eissa et al., 2026).

The diagnosis of the participants was retrieved from either inpatient or emergency hospital visits with stroke-related ICD10 codes. ICD-10 coding within the PHO is performed by trained clinical coders at the point of care, following standard national coding practice. Coded records undergo routine internal quality checks by MOH health information management teams, including logical-consistency checks (e.g., diagnosis codes checked against age, sex, and visit type for plausibility) and periodic review of coding completeness across contributing facilities. These checks are part of standard PHO data governance and were not designed or conducted specifically for this study. This is acknowledged as a limitation, and independent validation of stroke case-finding algorithms within the PHO is recommended as a priority for future methodological work. Diagnoses underwent routine PHO checks, but the study did not independently validate codes by chart review or a clinical reference standard; diagnostic accuracy is therefore unknown. Records with incomplete demographic or diagnostic information were labeled as missing data and were then excluded from the sub-analyses. Longitudinal patient-level data were extracted; For the main descriptive cohort, this meant that each patient was counted once, based on their earliest recorded stroke-related diagnosis within the 2016–2025 study period; subsequent or recurrent stroke-coded encounters for the same individual during the study period were not treated as separate cases and were not analyzed independently. This approach was applied consistently to the incidence analysis (2020–2025), in which only each patient's first qualifying inpatient or emergency stroke diagnosis was counted as an incident event. As a result, this study characterizes the population of individuals with at least one registry-recorded stroke diagnosis, rather than the total number of stroke events or recurrences occurring in Saudi Arabia during the study period; recurrence rates and multiple-event patterns were outside the scope of this descriptive analysis.

The data including demographics (age, sex, and region), clinical indicators (BMI categories of risk profiles and visits), and comorbidity profiles (hypertension and dyslipidaemia, risk profiles, and visits), and comorbidity profiles such as hypertension and dyslipidaemia.

### Data processing

Utilizing the unified healthcare registries available to the PHO, a data model of those diagnosed with stroke was created, including their individual clinical records, such as visits, labs, diagnoses and demographic data. The data were processed and modeled using SQL and data harmonization techniques. No imputation was performed; available-case analysis was used, with each denominator reported explicitly, and no person was excluded from the overall cohort solely for a missing non-essential variable.

Body mass index values were extracted from routinely collected electronic health records when available. Missing BMI values primarily reflect incomplete routine clinical documentation rather than data processing errors. BMI analyses were therefore restricted to participants with available measurements. Patient data were made unique by cross-validating IDs using the national population registry. Any incomplete health records were identified and excluded from the sub-analyses. Data quality assurance procedures included automated range and logical-consistency checks, deduplication via national ID cross-validation, and exclusion of records with contradictory or implausible values (e.g., invalid dates, out-of-range BMI). These checks are performed routinely by the PHO data governance team as part of standard data pipeline management.

### Statistical analysis

Descriptive analyses were performed to summarize demographic characteristics, regional distribution, healthcare utilization, and clinical characteristics. Because the primary objective of this study was to provide a descriptive assessment of registry-recorded stroke burden, crude estimates are presented and age-standardization was not performed. No age standardization, temporal trend tests, subgroup tests, or multivariable models were performed in this descriptive analysis. Because the primary objective of this study was descriptive epidemiology and surveillance of registry-recorded stroke burden, inferential statistical modeling was considered beyond the predefined scope of this analysis. The data analyses by Onley using the Impala Cloudera distributed environment, national-level data were queried and processed via SQL. The distribution of registry-recorded stroke/TIA diagnoses across age, sex and region was summarized in Excel tables and graphical representations. The study provides a population-level descriptive overview.

## Results

The PHO identified 68,634 unique adults with a qualifying cerebrovascular diagnosis. Given that the adult population of Saudi Arabia in 2025 was 26,454,318, Table 1 presents the demographic characteristics of individuals with registry-recorded stroke/TIA diagnoses. The 10-year accumulated numerator divided by the 1-year (2025) denominator yields 0.26%; this is a crude contextual ratio, not point prevalence, period prevalence, or cumulative risk.

**Table 1:** Demographic characteristics of individuals with registry-recorded stroke/TIA diagnoses in Saudi Arabia.

Characteristic	Vital state	Registry-recorded cohort, *n*	Cohort percentage	KSA adult population, *n*	Registry-recorded ratio
Gender	Male	44,238	64.45%	16,933,132	0.26%
	Female	24,396	35.55%	9,521,185	0.26%
Nationality	Saudi	52,362	76.29%	13,159,139	0.40%
	Non-Saudi	16,272	23.71%	13,295,179	0.12%
Insurance state	Insured	19,158	27.91%	8,151,079	0.24%
	Uninsured	49,476	72.09%	18,303,239	0.27%
Residence	Urban	68,070	99.18%	26,380,980	0.26%
	Rural	564	0.82%	73,338	0.77%
Vital state	Alive	51,435	74.94%	25,715,233	0.20%
	Deceased	17,199	25.06%	739,085	2.33%

Figure 1 summarizes the analysis of gender and age group distribution, excluding 256 individuals due to a lack of accurate age data. The figure shows that most registry-recorded stroke/TIA diagnoses occur between the ages of 60 and 69, accounting for 23.27% of all diagnoses. In every age group, the male population dominates; in the 60–69 bracket, males represent 15.14% of the total population (10,393), while females represent 8.13% (5,577). Overall, the registry-recorded stroke/TIA cohort.

**Figure 1:**
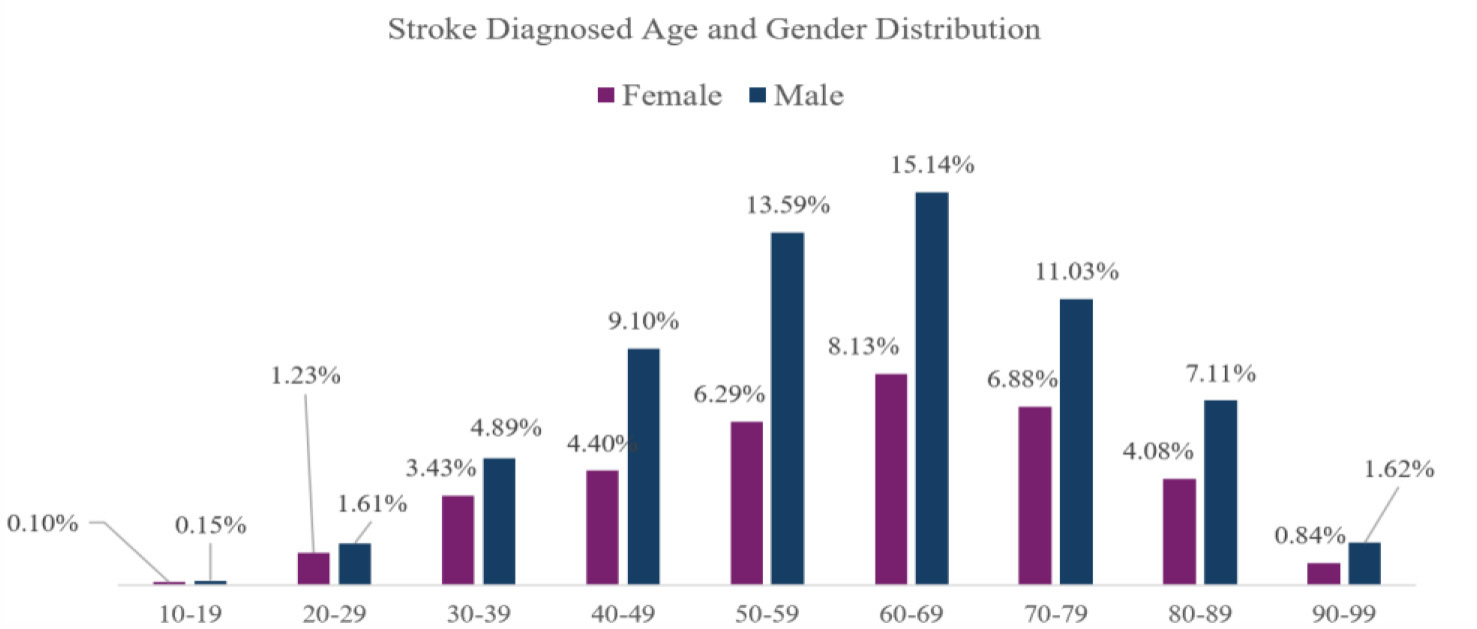
Age and gender distribution of individuals with registry-recorded stroke/TIA diagnoses in Saudi Arabia spanning from 10 to 99 years, where purple color represents females, and blue represents males.

These regional differences, including the crude regional registry-recorded ratios for all other regions, are shown in Figure 2. Crude cumulative registry-recorded proportions by administrative region (using 2025 regional adult-population denominators, not age-standardized) were highest in Najran (0.38%), Riyadh (0.31%), and Al Bahah (0.31%), and lowest in Tabuk (0.15%); ascertainment and registry coverage may contribute to these differences.

**Figure 2:**
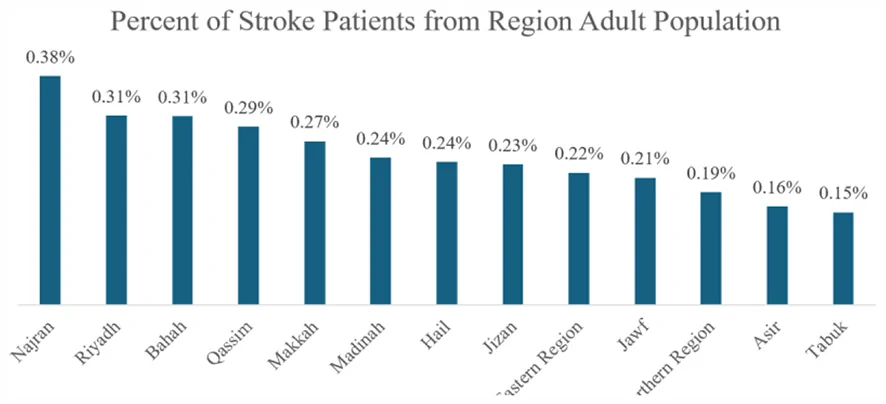
Crude regional ratios of individuals with registry-recorded stroke/TIA diagnoses among the 2025 adult population, Saudi Arabia, 2016–2025. The ratios use 2025 regional adult-population denominators and are not age-standardized. Differences may be influenced by regional variation in ascertainment, coding, and registry coverage.

Newly registry-recorded stroke/TIA diagnoses in Saudi Arabia between 2020 and 2025 are summarized in Figure 3. The figure is limited to these years due to a lack of data in prior years. Annual values reported here are counts of newly registry-recorded qualifying inpatient or emergency diagnoses, not incidence rates. The first diagnoses were based only on inpatient or emergency encounters; any other diagnoses, such as those in outpatient clinics, were excluded. The increase from 2020 to 2021 is due to an increase in data over time. Newly recorded counts were 4,762; 9,385; 12,155; 7,598; 7,763; and 7,753 for 2020–2025, respectively (total 49,416), increasing through 2022 and then similar in 2023–2025; these are counts, not incidence rates. The PHO counted each person once using the earliest qualifying inpatient or emergency diagnosis; recurrent encounters were not counted as additional cases.

**Figure 3:**
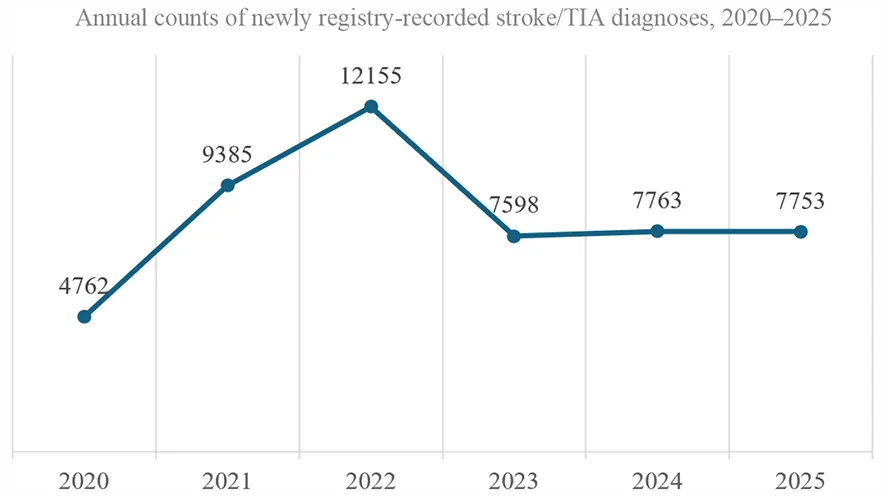
Annual counts of newly registry-recorded stroke/TIA diagnoses, 2020–2025.

Among individuals with a valid BMI measurement, overweight was the most frequent BMI category, making up 15.28% of the affected population. This was followed by those classified as obese (14.47%), healthy (12.01%) and underweight (0.71%). The PHO multimorbidity strata were defined according to the number of documented comorbid conditions in addition to the qualifying stroke/TIA diagnosis: low, no additional condition; rising, 1–2 additional conditions; high, exactly 3 additional conditions; and critical, 4 or more additional conditions. These categories were mutually exclusive and represent administrative multimorbidity strata rather than a validated cardiovascular-risk prediction score. In terms of healthcare service, More details about the weight categories, risk profile and visits are summarized in Table 2, Among 1,790,388 encounters with a recorded encounter type, outpatient visits accounted for 1,353,437 (75.59%), emergency visits for 326,479 (18.24%), and hospital admissions for 110,472 (6.17%). The ≥7-visit category included 1,552,895 encounters (86.61%) of the 1,792,921 encounters classified by visit-frequency category. Encounter-type analyses included 1,790,388 encounters with a recorded encounter type. The denominators therefore differ by 2,533 encounters because the availability and classification rules for the two variables were not identical. Most individuals fell into the PHO low-multimorbidity category (62.48%), followed by rising (31.32%), high (3,970; 5.78%), and critical (285; 0.42%) multimorbidity strata; these are administrative strata, not a validated cardiovascular-risk score.

**Table 2:** Body mass index (BMI) categories, comorbidity risk stratification and healthcare utilization among adults with registry-recorded stroke/TIA diagnoses.

Category	Subcategory	Stroke-diagnosed by category	Percent of stroke-diagnosed by category	All KSA count by category	Percent of KSA by category
BMI category	Obese	9,930	14.47%	3,204,094	0.31%
	Overweight	10,494	15.28%	3,426,615	0.31%
	Healthy weight	8,243	12.01%	6,128,910	0.13%
	Underweight	486	0.71%	4,611,555	0.01%
PHO multimorbidity stratum	Low	42,882	62.48%	7,648,012	0.56%
	Rising	21,497	31.32%	1,419,109	1.51%
	High	3,970	5.78%	116,977	3.39%
	Critical	285	0.42%	7,100	4.01%
Type of visit	Emergency Room Visits	326,479	18.24%	44,477,802	0.73%
	Outpatient Visits	1,353,437	75.59%	238,786,929	0.57%
	Hospital Admissions	110472	6.17%	7,662,577	1.44%
Category of visit	1-2 Visits	93,550	5.22%	15,953,030	0.59%
	3-4 Visits	78,623	4.39%	15,909,428	0.49%
	5-6 Visits	67,853	3.78%	14,853,540	0.46%
	7 or more visits	1,552,895	86.61%	253,472,648	0.61%

Those without any weight measurements or invalid weight measurements were removed from the table, comprising 57.55% of the participants. PHO multimorbidity strata were based on the number of documented comorbid conditions in addition to the qualifying stroke/TIA diagnosis: low, none; rising, 1–2; high, exactly 3; and critical, 4 or more. The categories were mutually exclusive and were not a validated cardiovascular-risk prediction score.

Among the 17,199 individuals recorded as deceased, 17,190 had sufficient information to calculate the elapsed time from the index registry-recorded stroke/TIA diagnosis to the recorded date of death; nine individuals had insufficient date information. Figure 4 presents the descriptive distribution of these elapsed-time intervals. The analysis was restricted to deceased individuals, follow-up was not uniformly defined, and censoring was not incorporated. Accordingly, these findings should not be interpreted as survival probabilities or mortality risks.

**Figure 4:**
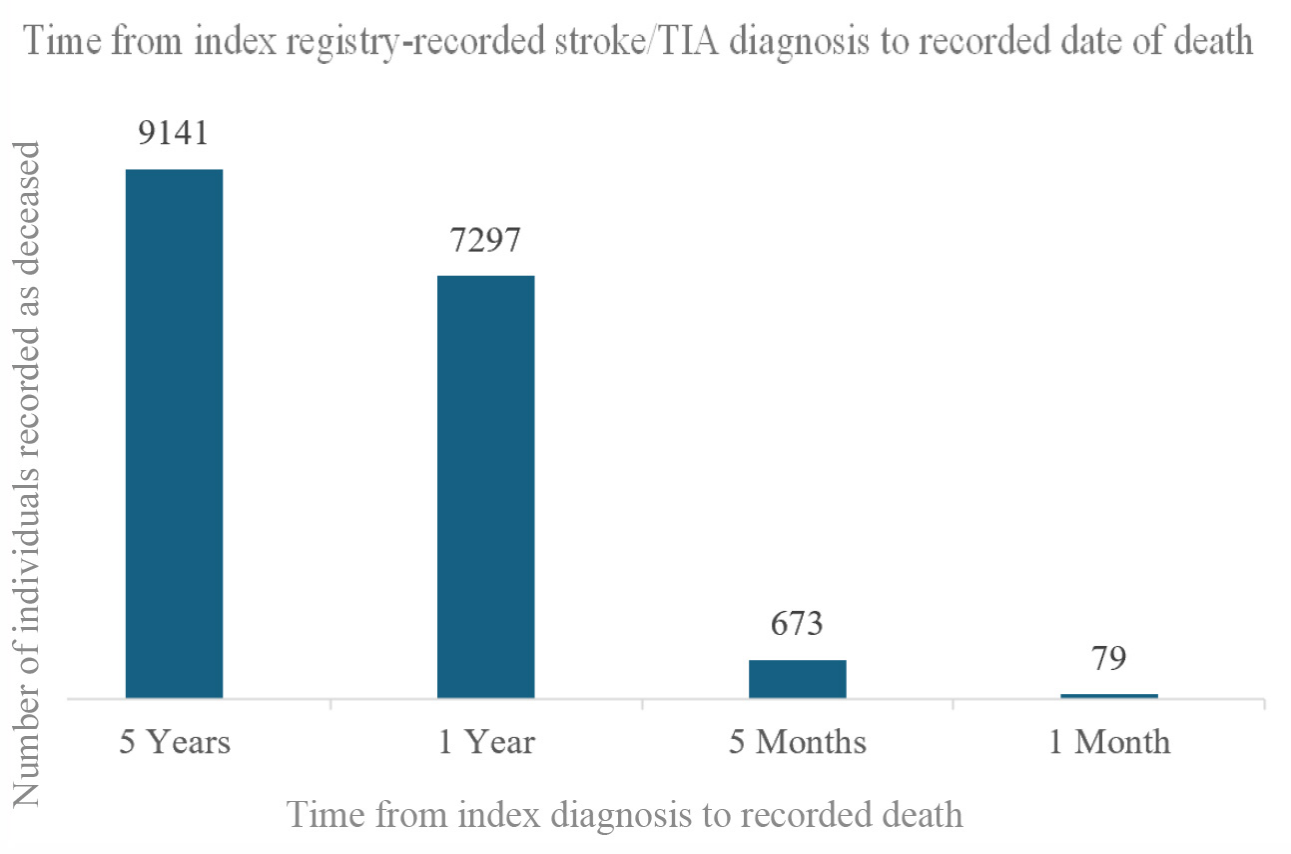
Descriptive distribution of the time from the index registry-recorded stroke/TIA diagnosis to the recorded date of death among individuals recorded as deceased, Saudi Arabia, 2016–2025. The analysis was restricted to deceased individuals and did not account for censoring; therefore, it does not represent a survival analysis.

## Discussion

Collectively, the results of this national study indicate a large yet disparate burden of stroke. In the PHO dataset, there were 68,634 individuals with registry-recorded stroke/TIA diagnoses among adults. The accumulated registry-recorded count corresponded to approximately 0.26% of the 2025 Saudi adult population. This is a crude contextual ratio and should not be interpreted as prevalence in PHO-integrated data over the study period. This value places Saudi Arabia in the range observed by previous national and regional surveys (MOH, n.d.; El-Hajj et al., 2016), and is significantly lower than the 6.50% prevalence of hypertension via the same PHO platform (Al Eissa et al., 2026) a difference which is indicative of the very different natural histories of the two diseases. Stroke is an acute, episodic process, and hypertension is a silent, cumulative process, which was only examined clinical.

The burden of stroke was disproportionately high among men almost two out of three cases and the age bracket with the highest risk was 60–69 years, representing 23.27% of all diagnoses. This trend is consistent with previous national and international literature (Feigin et al., 2024; Alenzy et al., 2026; Gou et al., 2025), and is clinically plausible. At this stage of life, decades of exposure to hypertension, dyslipidaemia and physical inactivity begin transforming into clinically observable cerebral events, which are further aggravated by the natural vascular aging processes.

The findings of the present study are consistent with recent international evidence demonstrating that stroke remains a leading cause of mortality and long-term disability worldwide. The Global Burden of Disease (GBD) 2021 study reported that although age-standardized registry-recorded proportions have declined in many countries, the absolute number of individuals with registry-recorded stroke/TIA diagnoses continues to increase, which may reflect of population growth, aging, and the rising prevalence of modifiable cardiovascular risk factors (Feigin et al., 2024). Similarly, the INTERSTROKE study identified hypertension, obesity, diabetes, dyslipidaemia, smoking, and physical inactivity as the principal contributors to stroke risk across diverse populations (O'Donnell et al., 2016). Furthermore, the American Heart Association and the European Stroke Organization continue to emphasize comprehensive cardiovascular risk reduction, early detection, and coordinated stroke systems of care as key priorities for reducing stroke burden. This study did not estimate risk-factor associations; the comparisons below are descriptive and limited by differing definitions, denominators, ascertainment, and age standardization across studies (Martin et al., 2025; Sandset et al., 2026). Future studies should evaluate the mechanism of missing BMI data and perform sensitivity analyses to assess the potential influence of missingness on obesity-related findings.

Comparable trends have been reported across Gulf Cooperation Council (GCC) countries, including the United Arab Emirates, Qatar, Kuwait, and Oman, where rapid demographic transition, an aging population, and a high prevalence of modifiable cardiovascular risk factors particularly hypertension, diabetes, obesity, and dyslipidaemia have similarly driven an increasing burden of stroke and a growing demand for prevention, acute management, and rehabilitation services (Feigin et al., 2024; El-Hajj et al., 2016; Alenzy et al., 2026; Deleu et al., 2006; Ganguly et al., 2021; Abujaber et al., 2024; Al-Hashel et al., 2016; Al-Shamsi et al., 2019; Mysore et al., 2026). GCC studies are used here as context only; differences in populations, definitions, and health systems preclude mechanistic equivalence with the present findings.

One of the most important findings of this study is the observed regional variation in registry-recorded stroke burden across Saudi Arabia, with Najran, Riyadh, and Al Bahah showing the highest proportions and Tabuk the lowest. These differences are likely multifactorial and should be interpreted cautiously. These are best described as crude regional ratios. Demographics, access, referral, coding, and data-capture maturity may all affect them (WHO, 2025; Jaberinezhad et al., 2022; Alenzy et al., 2026; Krishnamurthi et al., 2020). Differences in electronic health record maturity, coding practices, and data-capture completeness across regions may further contribute to reporting variation rather than true epidemiological differences. Because this study is based on routinely collected registry data, these explanations should be regarded as plausible hypotheses rather than causal determinants; future studies incorporating socioeconomic indicators and standardized data-quality measures are needed to clarify the true drivers of regional disparity.

The rural-urban difference in the rates of strokes is perhaps the most impressive finding of the demographic data. Rural dwellers represented only 0.82% of the stroke cohort, but their age-adjusted stroke registry-recorded proportion (0.77 %) was almost three times that of urban dwellers (0.26 %). This disparity may indicate structural impediments in rural communities, including limited access to preventive care, increased prevalence of uncontrolled hypertension and later access to care (Asirvatham and Marwan, 2014; Algahtani et al., 2024). They are not mere statistical oddities, but indicate a systematic disparity in the consequences of stroke, which health planning must directly address.

The most probable alternative explanation, before concluding that this spike reflects a real epidemiological trend, is that the observed apparent increase is due to the progressive growth and maturation of the national electronic health registry during this timeframe, rather than a true increase in stroke/TIA occurrence. This is a familiar effect in a registry-based study, with initial data tending to be a poor reflection of actual case numbers. Newly recorded counts increased through 2022 and were similar in 2023–2025; evolving PHO coverage prevents interpreting this pattern as an incidence trend. The observed increase in annual counts of newly registry-recorded diagnoses may reflect improvements in registry completeness, expansion of electronic health record coverage, and enhanced national data linkage rather than a true increase in stroke/TIA occurrence. Improvements in registry completeness, expansion of electronic health record coverage, and enhanced national data linkage over time rather than representing a true increase in stroke occurrence. Consequently, temporal trends should be interpreted with appropriate caution (Benchimol et al., 2015; Al-Senani et al., 2020). The overall trend, however, is still consistent with previous Saudi and regional estimates (El-Hajj et al., 2016; Alqahtani et al., 2020) and confirms the global picture that the absolute number of strokes is still increasing despite age-standardized rates slightly decreasing due primarily to increasing population and aging (Mahfouz et al., 2025).

BMI was missing/invalid for 57.52% of the cohort. Results based on BMI apply only to the measured subgroup and cannot establish an adiposity association. Consequently, obesity-related findings should be interpreted cautiously may reflect that individuals with available BMI measurements may not be fully representative of the entire cohort. These percentages support the already developed mechanistic connection between excess adiposity, hypertension, dyslipidaemia and the risk of ischaemic stroke (Wang et al., 2022; GBD 2016 Stroke Collaborators, 2019). The presence of a considerable number of missing BMI data (57.55) is a significant limitation and implies that these data should be regarded as indicative but not conclusive. However, they are very consistent with national statistics on the prevalence of hypertension (Aloufi et al., 2026), which suggest that they have a common underlying metabolic substrate that must be prevented.

These categories were derived from documented comorbidities recorded within the linked national administrative database rather than from comprehensive cardiovascular risk assessments. Therefore, the apparent predominance of the low-risk group is unlikely to represent the true absence of cardiovascular risk factors and may instead reflect incomplete documentation or under-coding of chronic conditions within routine clinical practice. Furthermore, important determinants of stroke risk, including smoking status, physical inactivity, dietary habits, family history, and other behavioral or socioeconomic factors, are not routinely captured in administrative ICD-10 datasets. Consequently, some individuals classified as low risk may in fact have significant unrecorded cardiovascular risk profiles. This limitation is inherent to studies based on routinely collected health data and should be considered when interpreting the observed risk distribution. Future improvements in electronic health record completeness and standardized documentation of cardiovascular risk factors will enhance the accuracy of multimorbidity classification and population-based stroke surveillance. Approximately 62% of patients were classified into the PHO low-multimorbidity category; incomplete diagnostic capture may understate cardiovascular risk.

Data on healthcare utilization portray a picture of long-term, high-intensity involvement in healthcare. Almost 87% of encounters were patient-initiated with seven or more documented contacts, and 75.59% of encounters with a recorded encounter type were outpatient visits. These trends are consistent with the endemic realities of post-stroke life, which include continuous rehabilitation, recurrence follow-up, management of secondary prevention and functional assistance that does not end when a patient leaves hospital care (Winstein et al., 2016). They also portend a significant and increasing burden on the healthcare infrastructure in Saudi Arabia, which is expected to escalate as stroke rehabilitation requirements rise with an aging population (Al-Senani et al., 2019; Alatawi, 2021; Alshakhs et al., 2024). These data are a strong argument for investing in structured, community-based, post-stroke care paths that can maintain patients throughout the entire spectrum of recovery, not only at the acute stage.

Among individuals recorded as deceased, the elapsed time between the index registry-recorded stroke/TIA diagnosis and the recorded date of death varied across the defined intervals. Because the analysis excluded individuals who remained alive, did not account for censoring, and was affected by variable follow-up duration, these results are descriptive and cannot be used to estimate survival probabilities, case fatality, or mortality risk following stroke/TIA. Previous studies have nevertheless demonstrated that stroke-related mortality and adverse outcomes are influenced by stroke characteristics, age, severity, prior disability, and multimorbidity (GBD 2016 Stroke Collaborators, 2019; Downer et al., 2024).

This study benefits from using one of the largest linked national healthcare datasets currently available in Saudi Arabia, enabling comprehensive evaluation of registry-recorded stroke diagnoses across all administrative regions. Nevertheless, several limitations should be acknowledged. As with all studies based on routinely collected administrative data, case ascertainment depends on the completeness and accuracy of ICD-10 coding and clinical documentation; under-reporting, coding variability, and misclassification may have resulted in incomplete or differential capture of stroke diagnoses across healthcare providers and regions, introducing potential information bias into regional comparisons and overall burden estimates (Benchimol et al., 2015). Several additional limitations warrant consideration. Incidence estimates were restricted to inpatient and emergency presentations, so registry-recorded stroke/TIA diagnoses first identified in outpatient settings were not captured, likely resulting in some underestimation (Krishnamurthi et al., 2020; Al-Senani et al., 2020). Missing BMI data (57.55% of the cohort) limit the representativeness of weight-related findings, and the apparent predominance of low-risk patients may reflect under-captured comorbidities rather than true clinical profile. Because the PHO was formally established in 2023, data completeness and consistency may vary across historical sources and time periods. Finally, as a descriptive registry-based study, this work can identify patterns but not establish causal relationships, and key lifestyle risk factors including smoking status and dietary habits are not captured in the underlying administrative data. Despite these limitations, the PHO remains a valuable and evolving national surveillance platform for population-level stroke burden estimation and policy planning (Benchimol et al., 2015).

### Future research opportunities

Future research should prioritize record-level longitudinal studies, case-algorithm validation, subtype-specific analyses, and linkage to severity, medication, and rehabilitation data, along with behavioral data collection. Future studies using the PHO database should incorporate age-standardized estimates, temporal trend analyses, subgroup analyses, and multivariable modeling to investigate factors associated with regional variation, healthcare utilization, and clinical outcomes. In addition, subtype-specific analyses of ischemic stroke, hemorrhagic stroke, and transient ischemic attack would further enhance understanding of cerebrovascular disease patterns in Saudi Arabia and support more targeted prevention and healthcare planning. Such studies will also require time-matched denominators, formal temporal methods, a reproducible PHO multimorbidity specification, and transparent evaluation of missingness and coverage.

## Conclusion

This national descriptive study characterizes individuals with registry-recorded stroke/TIA diagnoses captured in linked PHO data. It identifies demographic, regional, multimorbidity, and healthcare-utilization patterns but does not estimate population prevalence, incidence, survival probability, or causal associations. The findings may support national health-service planning and highlight the need to improve data completeness, coding standardization, diagnostic validation, and differentiation between stroke and TIA subtypes. Future research should use time-matched population denominators, age-standardized estimates, clearly defined follow-up periods, and appropriate longitudinal methods. Data completeness, validation, subtype separation, and age-standardized longitudinal analysis are identified as priorities for future work.

## Data Availability

The original contributions presented in the study are included in the article/Supplementary material, further inquiries can be directed to the corresponding author.

## Supplementary material

Supplementary Table S1 RECORD/STROBE reporting summary for this analysis of routinely collected health data.
